# Surgical Department

**Published:** 1877-10

**Authors:** 


### SURGICAL DEPARTMENT.

Service of Prof. Moses Gunn.

*Pott's Disease of the Spine.*

W. C. Irish, aged 22, a railroad laborer. This patient, in appearance robust and well nourished, of medium height, but stout and compact built, states that about four years ago he first had a pain in his back, without having received any injury that he can remember. At the same time he had pains in his chest and stomach, but having caught a slight cold paid no attention to them. About a year after this (the pains continuing), he noticed a prominence on the back bone at the seat of the pain. The pain increased very gradually, for he continued at his work, which required him to lift heavily, until last winter. Since then locomotion has become difficult and his gait unsteady, from disturbance of the nervous distribution, and as the recumbent posture gives him ease he has confined himself to bed. The prominence which you notice, and which gives an angularity to the spine, is about the size of a walnut and has remained stationary, so the patient states, during the past year. The patient's appetite is good, having been so from first to last. His father and mother are living and well, and no hereditary taint or predisposing dyscrasia can be discovered.

This is a rare case of Pott's disease of the spine, being somewhat out of the usual way, as the patient's health has remained good and he has worked a great part of the time. That circumstance tells us that his health and condition are prime, and that this cannot be a case originating in a scrofulous or vitiated constitution. If the patient had been scrofulous he would hardly have continued at his work, lifting heavily as it required. This rules out scrofula and throws us upon the probability of some mechanical origin.

I have frequently called your attention to the premonitory signs of pectoral and abdominal pains, as exhibited in this case, advising you that in a child who has these symptoms, which are often attributed to a cold, they should lead you, in the absence of pulmonary or abdominal lesion, to suspect disease of the spine.

The singular part of this case is that it should have gone on to softening of the bodies of the vertebræ, with so few of the usual signs and so little constitutional disturbance.

The patient, now for the first time, calls attention to his testicles which are swollen, but not tender. He says he first noticed the swelling coming on gradually about a year ago. This throws anything but light upon the subject, and gives a coloring of scrofula to the case.

He expresses himself as experiencing comparative ease when held up and the head and shouleers are braced back, from the throwing of weight off from the bodies and upon the articular process of the vertebræ—the hint upon which mechanical support is founded—and he is advised to wear the usual apparatus for these cases. His nutrition is so good that were it not for the swolllen testicles I would have no inclination to advise any medication, but as it is he will be put upon cod liver oil, iodine and tonics.

